# Correction: Liu et al. Optimization of Antibacterial Activity and Biosafety through Ultrashort Peptide/Cyclodextrin Inclusion Complexes. *Int. J. Mol. Sci.* 2023, *24*, 14801

**DOI:** 10.3390/ijms252312688

**Published:** 2024-11-26

**Authors:** Hang Liu, Lin Wang, Chen Yao

**Affiliations:** School of Chemistry and Chemical Engineering, Southeast University, Nanjing 211189, China; 220213312@seu.edu.cn (H.L.); 220203166@seu.edu.cn (L.W.)

In the original publication [1], there was a mistake in Figure 1c as published. A processed TEM image was uploaded due to insufficiently careful inspection. The corrected Figure 1c appears below. The authors state that the scientific conclusions are unaffected. This correction has been approved by the Academic Editor. The original publication has also been updated. 

## Figures and Tables

**Figure 1:**
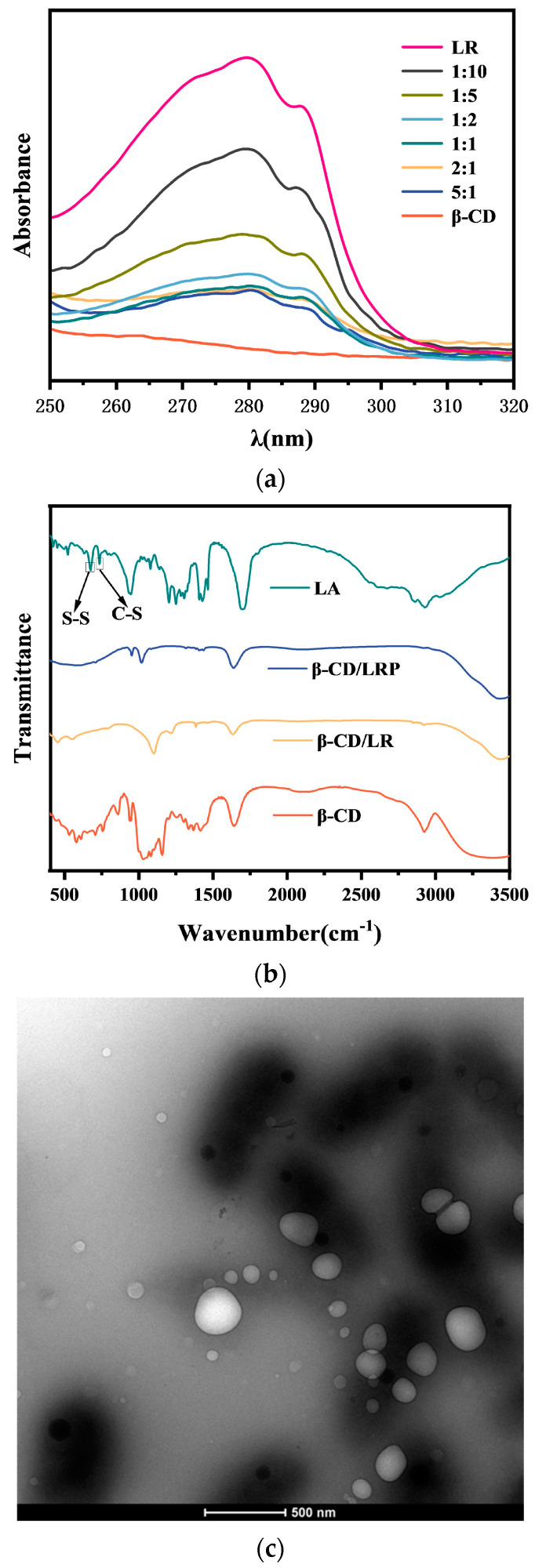
Characterization plots of inclusion complexes: (**a**) UV spectrogram of β–CD/LR; (**b**) IR spectra of β–CD/LR at 1:5 and β–CD/LRP at 1:3; (**c**) TEM plots of β–CD/LRP at 1:3.
